# Gold Nanoparticles Guided by Self‐Assembling Peptides: From Sequence to Superstructure

**DOI:** 10.1002/cbic.202500351

**Published:** 2025-07-21

**Authors:** Simone Adorinni, Silvia Marchesan

**Affiliations:** ^1^ Yusuf Hamied Department of Chemistry University of Cambridge Lensfield Road Cambridge CB2 1EW UK; ^2^ Department of Chemical & Pharmaceutical Sciences University of Trieste Via L. Giorgieri 1 34127 Trieste Italy

**Keywords:** AuNPs, AuNPs synthesis, nanocomposite materials, peptide hydrogels, self‐assembling peptides

## Abstract

Peptide‐based supramolecular assemblies have emerged as useful scaffolds to control the synthesis and spatial organization of gold nanoparticles (AuNPs) into nanocomposite superstructures. This concept article summarizes recent progress in the design of peptide‐directed AuNP assemblies, focusing on how subtle variations in peptide sequence, conjugation strategy, and synthesis conditions influence NP morphology, chiral arrangement, and the resulting optical properties. It highlights key strategies for rationally tailoring structural parameters, such as particle size, shape, and interparticle spacing, which determine the collective optical and plasmonic behaviors of the assemblies. The fundamental design rules discussed here provide critical insights for constructing programmable AuNP‐based nanomaterials, potentially useful in diverse fields including sensing, optical devices, and catalysis. Finally, it outlines current challenges and propose directions to further exploit the unique versatility of peptide‐nanoparticle hybrid systems in nanotechnology.

## Graphical abstract

Self‐assembling peptides act as versatile templates for gold nanoparticle (AuNP) synthesis. Their organization into discrete nanostructures or hydrogels enables control over AuNP morphology and assembly, offering new opportunities for functional nanomaterials.

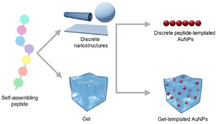

## 1 Introduction

Nanotechnology is reshaping the boundaries of innovation, offering unprecedented control over materials at the nanoscale (1–100 nm).^[^ ^1^ ^]^ The unique properties of nanomaterials arise from their small size, high surface‐to‐volume ratio, and quantum effects.^[^ ^2^ ^]^ Its impact spans from precision medicine, where nanoscale carriers deliver drugs directly to target cells,^[^ ^3^, ^4^ ^]^ to next‐generation electronics that enable faster and more efficient devices.^[^ ^5^, ^6^, ^7^ ^]^ In sustainability, nanomaterials enhance water purification^[^ ^8^, ^9^, ^10^, ^11^ ^]^ and energy storage,^[^ ^12^ ^]^ addressing critical global challenges. These applications demonstrate how nanotechnology is not only advancing scientific understanding but also driving innovation in diverse markets,^[^ ^13^ ^]^ redefining technological boundaries with profound societal implications.^[^ ^14^, ^15^, ^16^ ^]^

**Figure 1:**
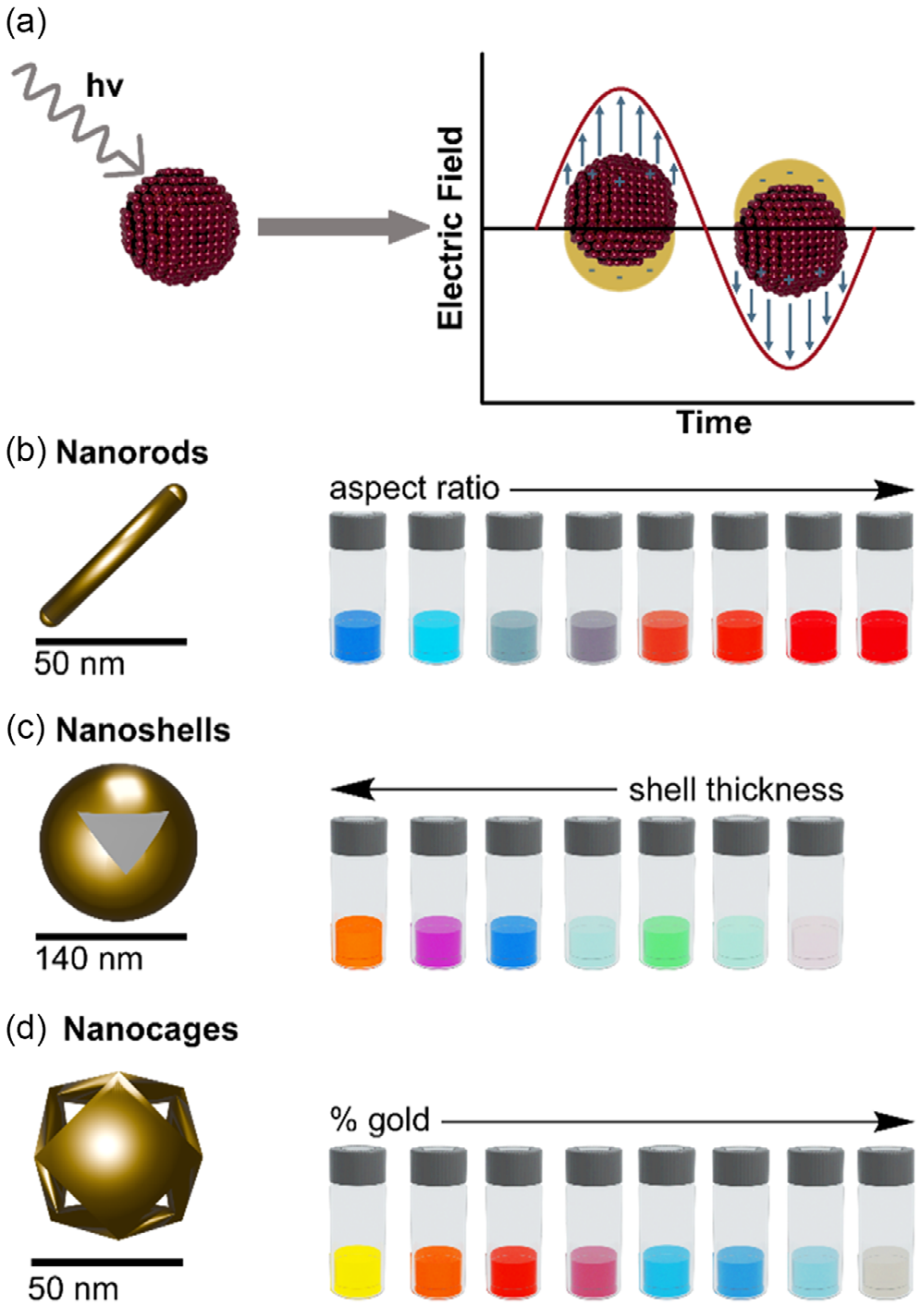
a) Graphical representation of localized surface plasmon resonance (LSPR) effect occurring on AuNPs. An incident wave excites the collective oscillation of free electrons. b–d) AuNPs of different shapes: (b) nanorods, (c) nanoshells, and (d) nanocages. The AuNPs exhibit striking colorations, due to the surface plasmon resonance, which causes photon absorption at specific wavelengths. These wavelengths shift based on factors such as aspect ratio, shell thickness, and/or gold‐induced galvanic displacement for nanocages.^[^ ^26^ ^]^

Today, there is a wide variety of nanomaterials differing in composition, morphology, dimensionality, and physical state.^[^ ^17^ ^]^ Among them, 0D metallic nanoparticles (NPs) have generated particular interest, owing to their extensive applications and the ability to synthesize them in numerous shapes and compositions.^[^ ^18^ ^]^ Metallic NPs exhibit unique optical and conductive properties, making them attractive for sensing,^[^ ^19^, ^20^ ^]^ imaging, electronics, and antimicrobial therapies.^[^ ^21^ ^]^ Such properties originate from their distinct localized surface plasmon resonance (LSPR),^[^ ^22^ ^]^ which arises when the oscillating electric field of incident light couples with the conduction electrons on the nanoparticle surface (**Figure** **1**a). Consequently, when LSPR occurs, metallic NPs strongly absorb photons that match their resonant frequency.

Gold NPs (AuNPs) display their LSPR peak in the visible region, which can be modulated by changing their size and shape (Figure 1b–d).^[^ ^23^ ^]^ This property combined with their chemical stability and ease of surface functionalization makes them extremely promising for a wide range of applications.^[^ ^24^, ^25^, ^26^ ^]^ Thus, synthesizing large quantities of highly monodisperse AuNPs is a key objective.^[^ ^27^, ^28^ ^]^

Combining bottom‐up strategies with supramolecular chemistry is particularly beneficial. Various supramolecular templates, ranging from simpler macrocycles^[^ ^29^ ^]^ and cyclodextrins^[^ ^30^ ^]^ to metal‐organic cages^[^ ^31^ ^]^ or mechanically interlocked molecules,^[^ ^32^ ^]^ can regulate AuNP growth through confinement or coordination. Gels also offer an intriguing platform by virtue of their mesh‐like network, allowing in situ AuNP synthesis.^[^ ^33^ ^]^ These soft materials vary in origin, from polymers^[^ ^34^, ^35^ ^]^ and metal‐organic frameworks^[^ ^36^, ^37^, ^38^, ^39^ ^]^ to woven architectures^[^ ^38^ ^]^ and metal‐organic cages.^[^ ^40^, ^41^ ^]^ In situ synthesis within gels yields hybrid systems harnessing the properties of both the soft scaffold and the metallic NPs.^[^ ^42^ ^]^

Peptides have emerged as versatile building blocks for supramolecular assembly, simultaneously facilitating biomineralization of metals.^[^ ^43^, ^44^ ^]^ By exploiting the broad range of natural and unnatural amino acids with diverse functionalities, peptides can self‐assemble into convenient functional scaffolds that can guide the nucleation and growth of AuNPs. This minireview examines how self‐assembling peptide‐assisted AuNP preparation can differ when peptides remain as discrete nanoassemblies or form extended nanostructures that entangle into macroscopic gels, discussing the pros and cons of each route. We also explore how distinct peptide functionalities determine AuNP size, shape, and stability.

## 2 Self‐Assembling Peptides as Templates

Self‐assembling peptides have been widely used to tailor AuNP formation and/or spatial arrangement at the nanoscale. There are several reasons that make these building blocks very attractive. Firstly, the literature is abundant with examples of self‐organizing sequences that yield discrete nanostructures, especially amyloid‐like fibrils, which could be decorated with AuNPs. Several noncovalent forces facilitate the binding between self‐assembling peptides and metal precursors, such as hydrogen bonding, electrostatic interactions, aromatic stacking, or gold–thiol coordination. A key early demonstration was conducted by Pochan and coworkers, who employed an alanine‐rich polypeptide, 17H6 (MGH_10_SSGHIHM(AAAQEAAAAQAAAQAEAAQAAQ)_6_AGGYGGMG), to form well‐defined fibrils with regularly spaced histidine patches.^[^ ^45^ ^]^ These histidine residues, exposed on the fibril surface, electrostatically immobilized negatively charged AuNPs. The polypeptide transition to β‐sheet fibrils required acidic pH (2.3) and elevated temperature, ensuring that the alanine‐rich segments engaged in hydrogen bonding and hydrophobic interactions. To reduce Au(III) ions into AuNPs, the system used sodium citrate and sodium 3‐mercaptopropionate. The histidine patches then acted as cationic binding sites, generating organized 1D nanoparticle arrays.

**Figure 2:**
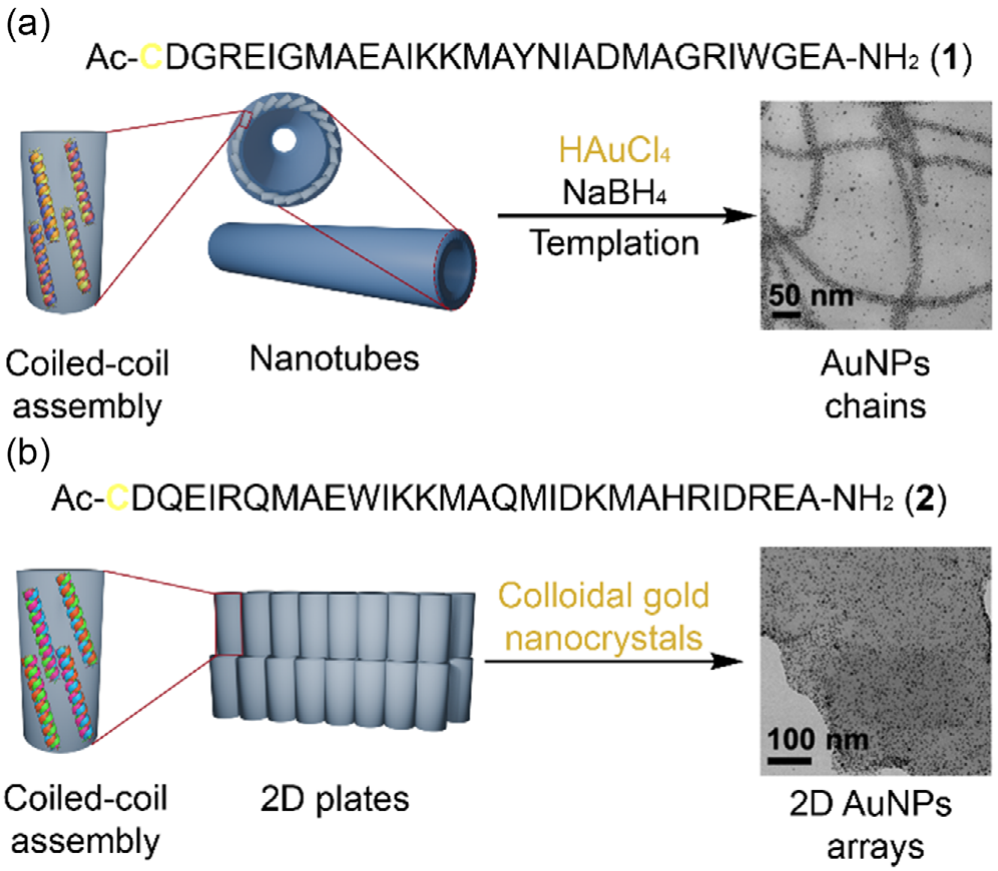
a) Ac‐CDGRIEGMAEAIKKMAYNIADMAGRIWGEA‐NH_2_ (**1**) self‐assembled into nanotubes templating the formation of AuNPs chains. b) Ac‐CDGRIEGMAEAIKKMAYNIADMAGRIWGEA‐NH_2_ (**2**) self‐assembled into 2D plates templating the formation of 2D AuNPs arrays.^[^ ^46^ ^]^ TEM micrographs adapted with permission.^[^ ^46^ ^]^ Copyright 2018, American Chemical Society.

Subsequent work by the same group in 2018 introduced cysteine residues into designed peptides Ac‐C DGRIEGM AEAIKKM AYNIADM AGRIWGE A‐NH_2_ (**1**) and Ac‐C DQEIRQM AEWIKKM AQMIDKM AHRIDRE A‐NH_2_ (**2**).^[^ ^46^ ^]^ These two peptides were able to form α‐helices characterized by a repetitive pattern of seven amino acids, called a heptad *(abcdefg)*_*n*_. This heptad pattern is typical of α‐helices that assemble into coiled‐coils.^[^ ^47^ ^]^ Within each heptad, the side chains at positions *a* and *d* are typically hydrophobic and contribute to the formation of an internal hydrophobic core that drives helix‐helix association, while residues at positions *e* and *g* often carry a charge and promote interhelical salt bridges that stabilize the overall bundle.^[^ ^48^ ^]^ In this case, the 30‐residue sequence includes four complete heptads within a Cys at the N‐terminus and an Ala at the C‐terminus. A total of eleven amino acids are directly responsible for mediating coiled‐coil formation. Specifically, the residues Ile5, Ala9, Ile12, Ala16, Ile19, Ala23, Ile26, and the C‐terminus Ala29 occupy positions *a* and *d* forming the hydrophobic core. The remaining three residues, Met8, Met15, and Met22, are located at position *g* in three successive heptads and further contribute to helix packing through their polar character. The remaining 18 amino acids were designed in the context of forming nanostructured materials, with the addition of one cysteine residue for the formation of AuNPs. Both **1** and **2** were able to form nanostructures, i.e., nanotubes and 2D plates, respectively (**Figure** **2**).^[^ ^46^ ^]^ Under slightly different conditions (pH 4.5 for nanotubes and pH 7 for plates), the cysteine thiol groups, whose oxidation to disulfide was prevented by the addition of tris(2‐carboxyethyl)phosphine, played a pivotal role in binding to gold precursor species and driving organized nanoparticle growth. The authors compared two experimental strategies. An in situ approach using HAuCl_4_ and NaBH_4_ for direct gold nucleation (Figure 2a), and a second approach that employed preformed gold colloidal nanocrystals (Figure 2b). In both cases, the presence of thiol groups was crucial for producing templated AuNP arrays; when cysteine was omitted, no organized arrays were observed, despite the formation of the corresponding supramolecular nanostructures.

**Figure 3:**
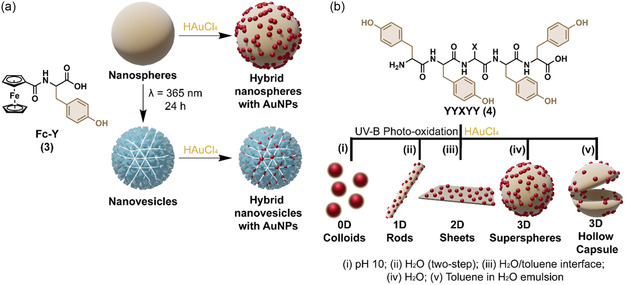
a) Fc‐Y (**3**) able to self‐assemble into nanospheres that can be transformed into nanovesicles by UV irradiation. Both the superstructures are able to act as reactor for the templated synthesis of AuNPs.^[^ ^49^ ^]^ b) YYXYY (**4**) able through UV irradiation to template the formation of different peptide nanostructures coated with spherical AuNPs, by changing the solvent conditions.^[^ ^51^ ^]^

Ferrocene–tyrosine (Fc–Y, **3**, **Figure** **3**a) adopts a β‐sheet secondary structure and can spontaneously self‐assemble, driven by backbone hydrogen bonding together with cooperative *π*−*π* stacking and hydrophobic interactions, into uniform nanospheres with different average diameters depending on the assembly conditions, either in darkness (480 nm) or under visible light (663 nm).^[^ ^49^ ^]^ Upon ultraviolet (UV) exposure, Fc–Y is first oxidized to its orthoquinone form, which then undergoes stepwise polymerization into oligomers (di‐, tri‐, and tetra‐Fc–Y). The authors propose that these oligomers nucleate on the surface of the nanospheres. Continued UV irradiation drives the remaining **3** monomers from the sphere core to the surface, where they also undergo oxidation and polymerization, transforming the solid nanospheres into hollow, rough‐surfaced nanovesicles (Figure 3a). Quinone formation disrupts the extended hydrogen‐bond network that sustains the β‐sheet secondary structure, and the peptides adopt β‐turn‐rich conformations. These shorter and flatter segments, together with covalent crosslinks and enhanced *π*−*π* stacking of ferrocene/quinone rings, pack laterally into lamellar sheets that constitute the vesicle shell. Both peptide nanospheres and nanovesicles reduce Au(III) to Au(0) without the addition of any external reducing agent, giving hybrid assemblies in which spherical AuNPs decorate the peptide self‐assembled nanostructures (Figure 3a).^[^ ^50^ ^]^ Moreover, the supramolecular peptide nanostructure controlled the size of the spherical AuNPs, which averaged 16.3 and 14.4 nm for nanospheres and nanovesicles, respectively.

In a similar way, Min and coworkers employed a tyrosine‐rich peptide (YYXYY, **4**, Figure 3b) to reduce Au(III) while simultaneously promoting self‐assembly under UV irradiation, resulting in hybrid nanostructures composed of AuNPs embedded within peptide assemblies.^[^ ^51^ ^]^ This peptide is highly versatile and can adopt different nanostructures depending on the solvent conditions. At pH 10 or in the presence of high DMSO content, the peptide remains well solvated and monomeric, adopting the characteristic right‐handed alpha‐helical conformation of tyrosine‐rich sequences. Despite remaining in its monomeric form, peptide **4** retains its ability to reduce Au(III), leading to the formation of spherical colloidal AuNPs surrounded by an organic peptide shell ≈1.86 nm thick. In aqueous environments or in the presence of low DMSO content, UV irradiation induces a conformational change to a random coil structure, which triggers self‐assembly into 3D superspheres (≈200 nm) that encapsulate AuNPs. This organization is driven by a combination of *π*−*π* interactions and hydrophobic effects. When the central residue X is a nonpolar amino acid, or either Asp or Asn, the peptide alone adopts a beta‐sheet conformation. In water, *π*−*π* stacking and hydrophobic interactions promote close contact between peptide molecules, resulting in directional growth into 1D nanorods. The subsequent addition of HAuCl_4_ and UV exposure results in the deposition of spherical AuNPs on the surface of the preformed peptide structures. This demonstrates that mineralization can also occur after the initial peptide organization. Solvent composition has a significant influence on the resulting morphology. In a toluene–water mixture, the peptide assembles at the liquid–liquid interface to form 2D nanosheets. Although insoluble in toluene, the peptide interacts with it through hydrophobic contacts and aromatic packing. These interactions are thought to cause local accumulation of peptide at the interface, promoting lateral assembly into extended nanosheet structures. In contrast, the use of a water–toluene emulsion leads to the formation of 3D hollow nanocapsules. Initially, the peptide coats the surface of the emulsion droplets as a 2D layer. After toluene removal, this shell collapses, yielding empty nanospheres. These findings highlight the key role of tyrosine residues in directing both the reduction of Au(III) and the structural outcome of the assembly process. The ability of these peptides to organize into defined nanostructures provides a confined environment that acts as a template for AuNP formation.

**Figure 4:**
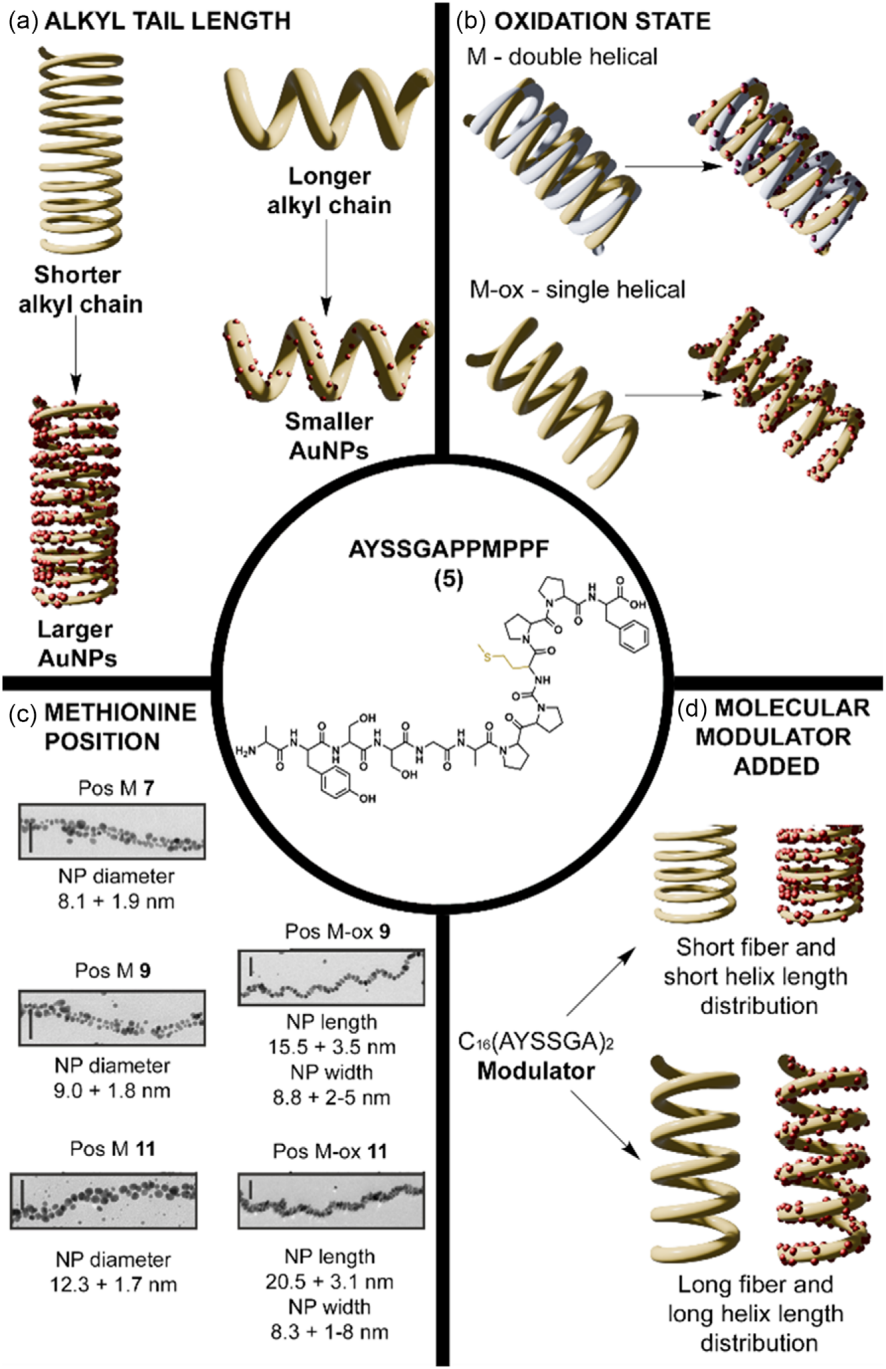
In the middle the structure of the generic peptide AYSSGAPPMPPF (**5**). a) The illustration showed the effect of the chain length on the helical pitch and the AuNPs dimensions.^[^ ^55^, ^57^ ^]^ b) The illustration showed that the oxidation of the Met directs the assembly of gold nanoparticle single helices, instead of double helices as happened for the unoxidized Met.^[^ ^56^ ^]^ c) The methionine position influences the dimensions of the resulting single and double helices AuNPs. In the high‐magnification TEM images, the scale bar corresponds to 50 nm.^[^ ^58^ ^]^ d) The addition of a molecular modulator, C16‐(AYSSGA)_2_, influenced the helix length distribution, promoting the nucleation.^[^ ^59^ ^]^ TEM micrographs adapted with permission.^[^ ^58^ ^]^ Copyright 2019, American Chemical Society.

The Rosi group has extensively explored peptide‐based strategies for directing the self‐assembly of gold nanoparticles (AuNPs) into chiral architectures. Central to this methodology is the rational design of peptide conjugates that both bind to AuNP surfaces and organize into supramolecular scaffolds.^[^ ^52^ ^]^ The peptide chosen was AYSSGAPPMPPF (referred to as PEPAu, **5**, **Figure** **4**), selected for its high affinity for gold surfaces. A key residue in the sequence is methionine (Met), which interacts strongly with gold and can be oxidized to methionine sulfoxide (M‐ox, **5**_**ox**_), providing an additional means of modulating the assembly behavior. The peptide can also be functionalized with alkyl chains of different lengths (**C**_**n**_), which strongly influence the structure and morphology of the helical assemblies. The first example of this assembly strategy involved the peptide‐conjugate molecule **C**_**12**_ **‐5**.^[^ ^53^ ^]^ In HEPES buffer, **C**_**12**_ **‐5** formed fibers with a twisted, left‐handed nanoribbon morphology. The peptide adopts a β‐sheet secondary structure, in which the formation of parallel β‐sheets and favorable hydrophobic interactions between the aliphatic tails drive the longitudinal organization of **C**_**12**_ **‐5** along the nanoribbon axis. Under these same self‐assembly conditions, the reduction of Au(III) occured via a mechanism involving the Tyr residue and the HEPES buffer. This led to the formation of AuNPs decorating the ribbon surface and forming double‐helical AuNP structures.^[^ ^53^ ^]^

Small modifications to the alkyl segment can dramatically affect the final architecture. For instance, changing the tail from **C**_**12**_ to **C**_**6**_ disrupts self‐assembly, resulting only in individual AuNPs or their aggregates.^[^ ^54^ ^]^ The propensity of **C**_**n**_ **‐5** to assemble into fibers is dictated by the relative ratio of its hydrophobic (aliphatic tail) and hydrophilic (peptide) components. Increasing the length of the aliphatic tails promotes fiber formation.^[^ ^55^ ^]^ However, if the aliphatic tails are too short relative to the size of the peptide head group, the conjugates do not assemble into fibers. This balance between tail and head group can be tuned by adding up to three peptide moieties, a parameter referred to as valency. In such cases, the conjugates instead form spherical or aggregated nanostructures that maximize hydrophobic interactions. At the same time, increasing the peptide valency reduces the overall hydrophobicity of the conjugate, thereby lowering the driving force for self‐assembly. As a result, only mono‐ and divalent conjugates are able to assemble into fibers. The monovalent conjugates form fibers with a greater width, attributed to more favorable side‐by‐side packing and stronger hydrophobic interactions. Fiber width was not affected by the tail length, although the tail length influences the helical pitch, which increases with longer chains. In contrast, increasing peptide valency leads to a decrease in pitch. The structural parameters of the AuNP superstructures, such as interchain distance and helical pitch, closely match those of the conjugate soft assemblies, highlighting the importance of the peptide templating effect (Figure 4a). Conjugates with higher valency also result in smaller AuNPs due to increased binding affinity. The propensity for fiber formation is also crucial. When the propensity for self‐assembly is higher, larger AuNPs are formed because the peptides have less time to cap the nanoparticle growth.

A key finding of this study is that the oxidation state of methionine significantly influences the morphology of the resulting peptide assemblies. Oxidation of the methionine residues in the divalent conjugate **C**_**18**_ **‐(5)**_2_ leads to the formation of fibers that adopt a helical ribbon configuration, rather than a twisted ribbon morphology. Helical ribbons display cylindrical curvature, with one surface facing the interior of the coil and the opposite surface exposed to the environment.^[^ ^56^ ^]^ Within these ribbons, **C**_**18**_ **‐(5**_**ox**_) aligns perpendicular to the ribbon plane, consistent with a cross‐β structural motif. This arrangement exposes the polyproline II helices and the deprotonated carboxylate groups (at near‐neutral pH) on the outer surface of the ribbon. Rod‐shaped AuNPs bind to these exposed regions, resulting in the formation of single helices whose pitch closely matches that of the peptide‐based scaffold (Figure 4b). Variation in the alkyl chain length produced peptide nanostructures that resembled those formed by the unoxidized Met peptide.^[^ ^55^, ^57^ ^]^ However, the dimensions of the resulting AuNPs differed considerably. Peptide conjugates with longer alkyl chains assembled more rapidly into closed helical ribbons, which led to the formation of smaller, spherical AuNPs.^[^ ^57^ ^]^ In contrast, shorter alkyl chains formed twisted ribbons more slowly, allowing nanoparticles more time to grow and resulting in larger, rod‐like shapes. This behavior indicates that the kinetics of peptide assembly, which are influenced by the hydrophobicity and length of the alkyl tail, can indirectly control nanoparticle size and shape by affecting the time available for growth before incorporation into the structure (Figure 4a).

A systematic investigation into the positional effects of Met within the **5** sequence demonstrated that moving the Met or M‐ox residue from position 7 to position 11 led to measurable differences in nanoparticle size, aspect ratio, and chiroptical intensity of the final superstructure (Figure 4c).^[^ ^58^ ^]^ Model studies using peptide **5** without any alkyl chain revealed that placing Met at position 7 or 9 produced AuNPs with diameters of 6.4 and 6.7 nm, respectively. Larger AuNPs (14.5 nm) were observed when Met was positioned at residue 11. In the former case, the thioether side chain is close to the rigid proline segment, enabling strong gold binding and favouring the formation of smaller AuNPs. When Met is at position 11, the side chain is distant from the proline recognition motif, weakening the interaction. Oxidation of Met reduces the intrinsic binding energy by half, resulting in larger AuNPs or aggregates when Met is at position 11. Subsequent investigations focused on the effect of Met position on the organization of AuNPs within peptide‐based helical superstructures. For unoxidized Met, the overall morphology was consistent with previous studies. Interestingly, the helical pitch (≈91–93 nm) remained constant regardless of Met position. For Met‐ox, AuNP single helices were observed only when Met was at position 9 or 11, with the latter yielding larger nanoparticles.

The Rosi group showed that assembly outcomes can also be modulated by introducing a short, secondary peptide, C_16_‐(AYSSGA)_2_, into the synthesis (Figure 4d).^[^ ^59^ ^]^ This molecular modulator lacks the gold‐binding domain but retains the β‐sheet‐forming region, allowing it to co‐assemble with the primary conjugate. Its incorporation accelerates nucleation while limiting fiber elongation, resulting in the formation of shorter helices with narrower length distributions. In the absence of the modulator, the assemblies grow into longer, more polydisperse fibers. By varying the proportion of the modulator relative to the full‐length peptide, it is possible to achieve precise control over the final helix dimensions. This strategy demonstrates how targeted manipulation of assembly kinetics can improve structural uniformity in chiral nanoparticle systems, without requiring changes to the core molecular architecture. This approach underscores that not only individual peptide design, but also co‐assembly strategies, are powerful tools for achieving structurally precise chiral nanomaterials.

Beyond structural control, this tunability also influences the stability of the resulting materials. Increasing the size of the gold nanoparticle components enhances both thermal and chemical resistance. Helical assemblies composed of larger nanoparticles preserve their morphology at elevated temperatures and remain intact in denaturing conditions such as high concentrations of urea or in the presence of proteolytic enzymes including proteinase K.^[^ ^60^ ^]^ These findings indicate that nanoparticle dimensions can be adjusted not only to control structural and optical features but also to improve material robustness, which is particularly important for applications that require environmental or biological compatibility.

## 3 Peptide Gels as Soft Templates

Peptide gels provide a 3D matrix that can serve as a convenient solid scaffold to confine AuNPs within a 3D macroscopic yet nanostructured soft material, which, by definition, contains a large amount of solvent, thus can be exploited as a nanoreactor. However, reactants and products diffusion within gels can be a challenge, thus limiting the efficiency of reactions carried out within such solid scaffolds, as well as posing issues related to heterogeneity of local concentrations. For these reasons, mastering the use of peptide gels as reactors requires fine‐tuning of their viscoelastic properties. Furthermore, if the peptide gelators actively participate as reagents in the AuNP formation, it is possible they lose their self‐assembling ability, thus leading to the disruption of the gel. Nevertheless, careful control over experimental conditions led different research groups to positive outcomes for AuNP formation.

Banerjee and coworkers developed organogels from tripeptides containing tyrosine, achieving AuNP formation without external reducing agents.^[^ ^61^ ^]^ Boc‐FYF and Boc‐YFY require triethylamine (TEA) to oxidize the tyrosine phenol at higher temperatures,^[^ ^62^ ^]^ while Boc‐YYF can form a hydrogel under slightly basic conditions (pH 10) and reduce HAuCl_4_ directly. Such variations highlight how choice of peptide sequence and solvent environment can control nanoparticle placement, alignment, and size.

**Figure 5:**
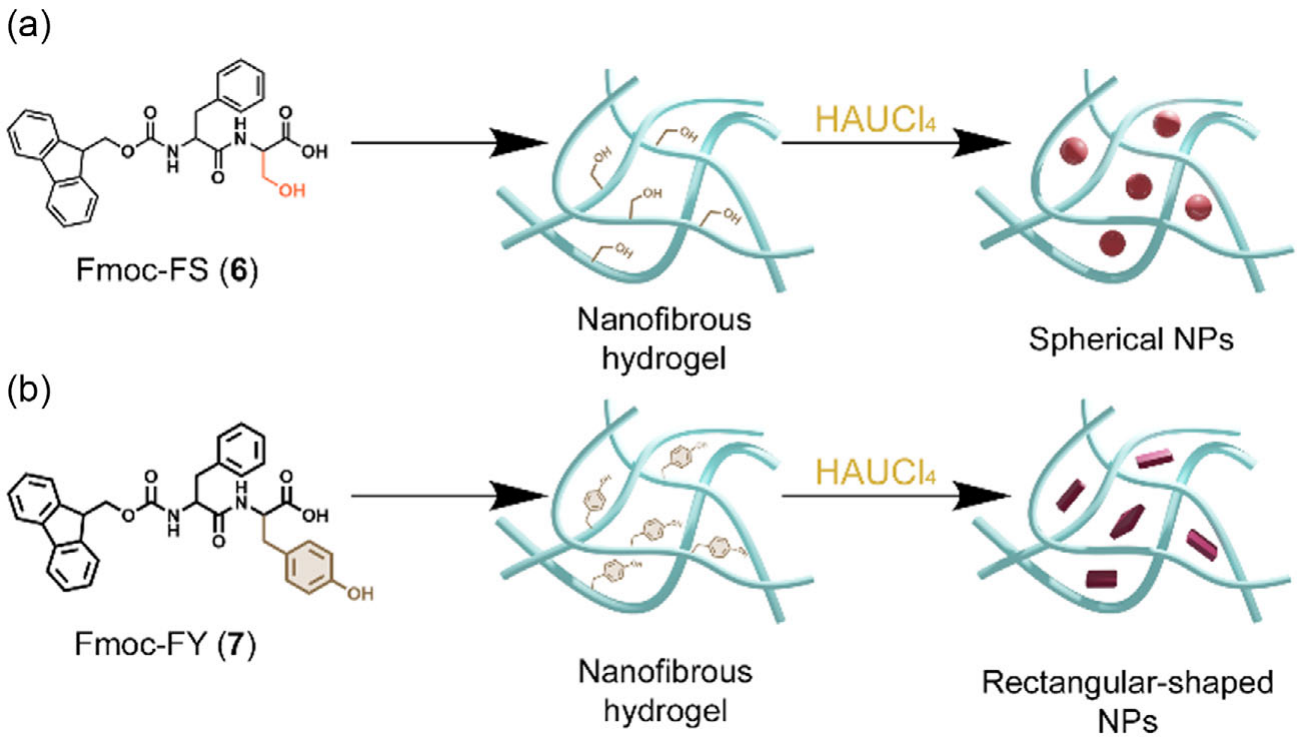
a) Fmoc‐FS (**6**) self‐assembled in nanofibrous hydrogel able to template the formation of spherical AuNPs under a thermodynamic pathway. b) Fmoc‐FY (**7**) self‐assembled in nanofibrous hydrogel able to template the formation of rectangular AuNPs under a kinetic pathway.^[^ ^63^ ^]^

Hydroxyl groups are effective at accumulating and reducing Au(III) ions; therefore, amino acids such as tyrosine and serine are well‐suited for designing gels that can template AuNP formation. In 2019, Roy and coworker developed a series of different dipeptides containing a N‐terminus Phe and Tyr or the Ser (**Figure** **5**).^[^ ^63^ ^]^ The dipeptides were functionalized with different N‐capping groups (Fmoc, Nap, and Cbz) to study how the alteration in size and hydrophobicity of the aromatic capping influence the gelation process. These peptides have been tested for a shape‐controlled synthesis of AuNPs. The different pH plays a key role in the shape control process. At higher pH (≈11), the reduction occurs more rapidly, yielding smaller nanoparticles, whereas at lower pH (≈8), the slower reduction process favors the formation of larger particles. In the case of the serine‐based gelator (**6**), the hydroxyl group promotes thermodynamically favored growth, resulting in spherical nanoparticles (Figure 5a). While in the case of the tyrosine‐based gelator (**7**), the synthesis follows a kinetically controlled pathway that produces rectangular‐shaped nanoparticles (Figure 5b).

Tryptophan represents another effective amino acid for the design of peptide‐based hydrogels capable of templating the in situ synthesis of AuNPs.^[^ ^64^, ^65^ ^]^ The redox‐active indole N–H moiety can reduce HAuCl_4_ without external reductants, while *π*‐stacking and hydrogen bonding interactions contribute to nanoparticle stabilization within the hydrogel network.^[^ ^64^ ^]^ The distinct morphologies of the hydrogel structures, governed by peptide sequence and assembly conditions, direct the formation of shape‐specific AuNPs, including triangular sheets, wires, and polyhedral nanocrystals.^[^ ^64^ ^]^ This shape control arises from the ability of the hydrogel to modulate the surface energies of specific Au crystal facets, without the need for seed‐mediated growth, which otherwise leads to irregular shapes.^[^ ^64^ ^]^ Interestingly, when the indole redox activity was chemically suppressed by N‐methylation, Au(III) reduction efficiency decreased, yet spherical AuNPs could still form, suggesting that other functional groups, such as carboxylate and aromatic residues, synergistically contribute to the reduction process.^[^ ^65^ ^]^ Systematic simplifications confirmed this cumulative effect, highlighting the complex, multisite nature of Au(III) reduction within these peptide‐based systems.^[^ ^65^ ^]^

**Figure 6:**
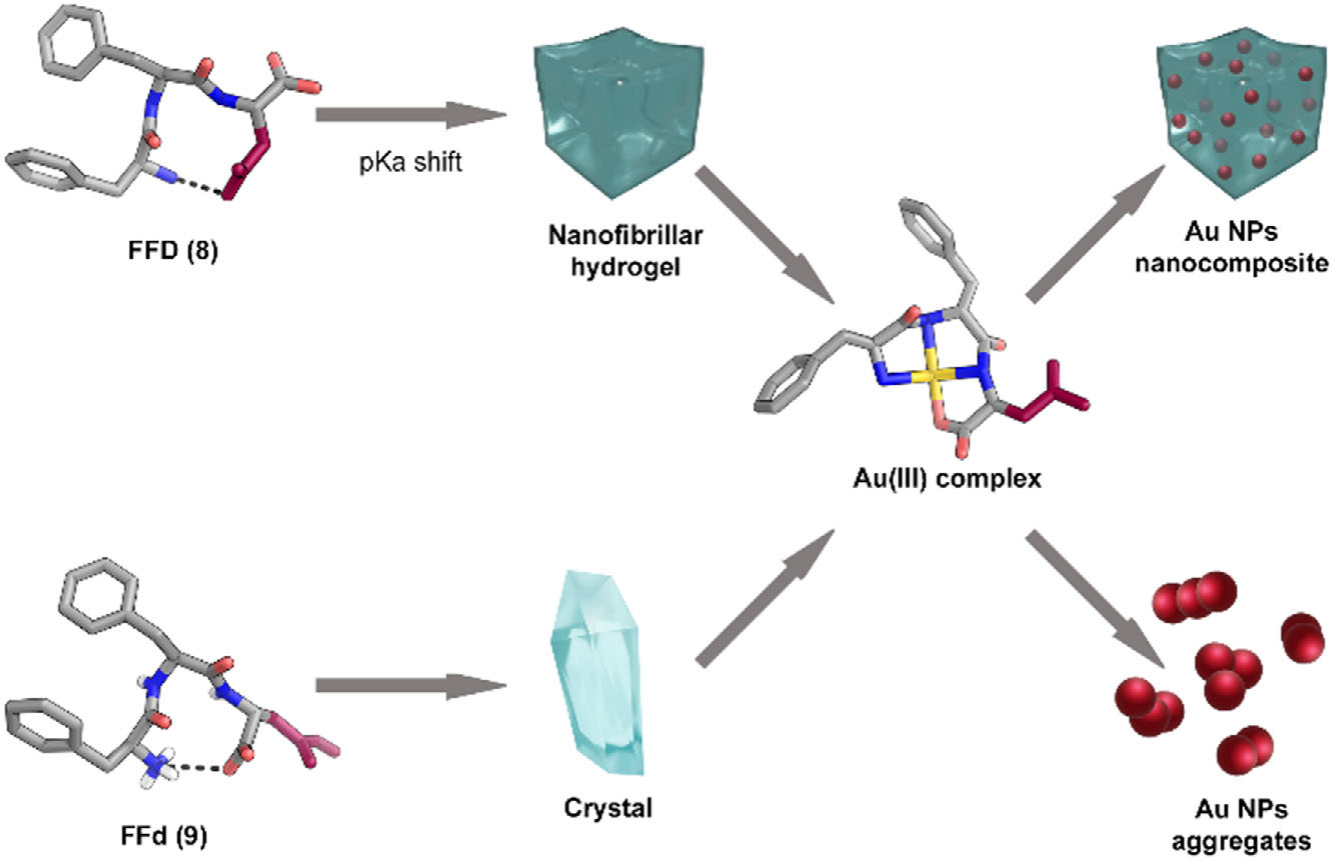
Self‐assembly behavior and Au(III) templating ability of two stereoisomeric tripeptides, FFD (**8**) and FFd (**9**), containing aspartic acid. The homochiral peptide **8** forms an intramolecular hydrogen bond that shifts the pK_a_, enabling gelation into a nanofibrillar hydrogel, which serves as a soft template for controlled AuNP formation. Both peptides can coordinate Au(III) via a square‐planar complex that reduces to Au(0), but only the hydrogel environment prevents aggregation, yielding well‐dispersed nanocomposites.^[^ ^66^ ^]^

In a work published in 2024, we and other collaborators developed tripeptides containing aspartic acid that can serve as a template for the synthesis of AuNPs.^[^ ^66^ ^]^ The study revealed that only the homochiral peptide (L‐Phe‐L‐Phe‐L‐Asp) was able to gel. Furthermore, XRD analysis revealed a different degree of disorder of these amphiphilic sequences in water according to the different stereoconfiguration of the α carbon. It was observed that only the homochiral peptide (**8**) and its stereoisomer L‐L‐D (**9**) could form an intramolecular hydrogen bond between the N‐terminal amine group and the carboxyl group of the side‐chain, for **8**, or the C‐terminus, for **9** (**Figure** **6**). The hydrogen bond was found to be responsible for a significant pKa shift, which enabled the homochiral peptide zwitterion to be the predominant species at pH 4–6, reaching the critical threshold necessary for gelation. All four peptides were able to complex Au(III), forming a square‐planar complex that is unstable in water (Figure 6). This complex undergoes oxidation to form a dehydropeptide, with contemporary reduction of the Au(III) to Au(0). It is interesting to note that previous report of similar tripeptide‐Au(III) complexes based on hydrophobic amino acids were stable in water, highlighting the key role of the Asp in the reactivity of Phe‐Phe‐Asp stereoisomers. Furthermore, the presence of the hydrogel was instrumental in regulating NP size, as all the peptides were capable of reducing the Au(III). However, in the absence of the hydrogel, the NPs aggregated (Figure 6). Therefore, this work demonstrated how AuNPs can be templated and grown in a green method within a nanostructured yet macroscopic soft material composed of a peptide, which served multiple roles: as scaffold, as mild reducing agent, and as a soft capping agent to avoid their aggregation, while leaving the AuNPs’ surface available and reactive as demonstrated in a proof‐of‐concept application (see next section).

## 4 Applications

Peptide‐mediated gold nanoparticle nanocomposites show promise for numerous applications, from catalysis and energy to sensing and medicine. By fine‐tuning peptide sequences, self‐assembly conditions, and redox interactions, these hybrids can be optimized for specific functionalities.

**Figure 7:**
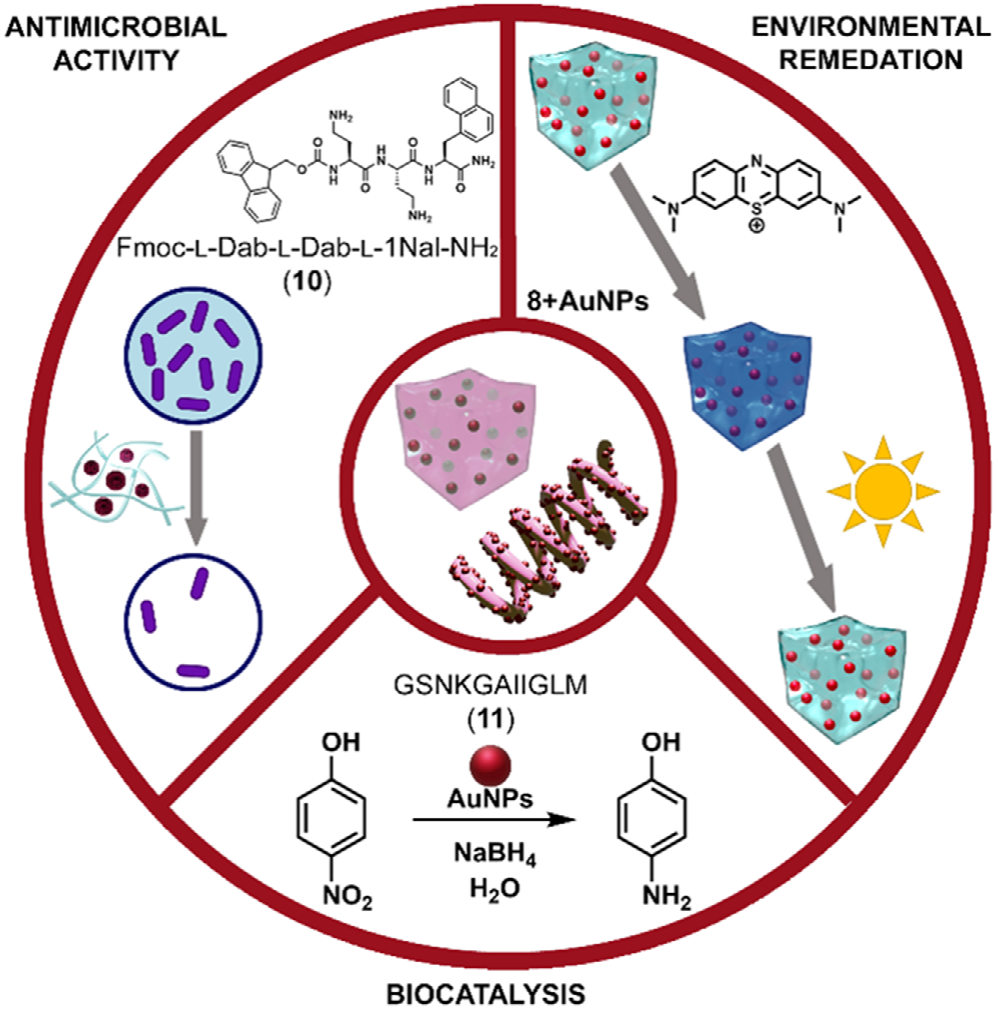
Peptide–AuNPs nanocomposites enable diverse applications through controlled self‐assembly. Rational design of short peptides allows for the formation of functional hydrogels hosting AuNPs. Heterochiral Fmoc‐peptide **10** forms AuNP‐loaded hydrogels with potent antibacterial activity.^[^ ^67^ ^]^ Amyloid‐derived peptide **11** guides the formation of catalytically active AuNPs for the reduction of 4‐nitrophenol.^[^ ^68^ ^]^ Nanofibrillar hydrogel formed by peptide **8** supports well‐dispersed AuNPs capable of degrading organic dyes for environmental remediation.^[^ ^66^ ^]^ Sequence‐driven control over nanoparticle morphology and peptide assembly underpins the multifunctionality of these hybrid systems.

Sarojini and coworkers reported that the combination of Fmoc‐L‐Dab‐L‐Dab‐L‐1Nal‐NH_2_ (**10**) and AuNPs formed in situ yielded hydrogels with potent antimicrobial properties and minimal cytotoxicity (**Figure** **7**).^[^ ^67^ ^]^ Variation in peptide sequences significantly changed the final NP geometry, while the hydrogel environment further modulated particle size and distribution. On the other hand, similar peptides sequence, Fmoc‐D‐Dab‐L‐Dab‐L‐1Nal‐OH and Fmoc‐D‐Dab‐L‐Dab‐L‐1Nal‐L‐1Nal‐L‐Dab‐OH, were also able to gelate and template the formation of AuNPs that did not show any antimicrobial activity, but excellent cytocompatibility. These systems can be extended to wound dressings or tissue engineering scaffolds where controlled particle release or local antimicrobial action is desirable.

**3**‐based AuNPs demonstrated enzyme‐like behavior, oxidizing glucose into gluconic acid and hydrogen peroxide, which in turn drives a colorimetric assay with tetramethylbenzidine in the presence of horseradish peroxidase.^[^ ^49^ ^]^ By altering the nanoscale morphology (nanospheres vs. vesicles), one can tune catalytic efficiency. Similarly, assemblies of amyloid‐β peptides GSNKGAIIGLM (**11**) can direct AuNPs into specific shapes (i.e., nanospheres, nanoribbons, nanofibres, and nanoflowers), each exhibiting distinct catalytic performances for reducing 4‐nitrophenol (Figure 7).^[^ ^68^ ^]^ Smaller NPs generally provide larger surface‐to‐volume ratios, enhancing catalytic rates.

These nanocomposites have shown to be particularly efficient also for environmental remediation. Stable hydrogels hosting well‐dispersed AuNPs can degrade organic dyes like methylene blue under mild conditions, as shown by Marchesan's group (Figure 7).^[^ ^66^ ^]^

Collectively, these applications highlight how rational peptide design, combined with meticulous control of NP synthesis and self‐assembly, yields versatile nanomaterials with tailored performance. The ability to achieve both hierarchical organization and multifaceted functionality is a key advantage of peptide‐directed AuNP systems.

## 5 Summary and Outlook

In conclusion, peptide‐directed assembly is emerging as a versatile and powerful strategy for synthesizing AuNPs with precisely controlled morphologies and distinct chiroptical properties. Two main approaches are commonly adopted, each offering unique advantages and outcomes in terms of NP control, processing, and application potential. In one approach, peptides self‐assemble into discrete nanostructures that act as templates to guide AuNP nucleation and spatial arrangement at the nanoscale. This method enables tight spatial control over nucleation sites, resulting in uniform interparticle distances, narrow size distributions, and well‐defined cluster geometries. Notably, chiral peptide assemblies can transfer their stereochemical information directly to the NP arrangement, enhancing plasmonic circular dichroism signals. In the second strategy, peptides form extended macroscopic networks, typically hydrogels, where AuNPs nucleate and grow in situ. These gels provide a continuous 3D scaffold that enables large‐scale organization and facilitates integration into bulk materials.^[^ ^42^ ^]^ However, diffusion‐controlled processes in hydrogels can introduce variability in particle size and distribution, particularly if reaction conditions are not carefully optimized. Tuning viscoelastic and transport properties is thus essential to ensure uniform growth and reproducibility.

While peptide‐based systems present compelling advantages, particularly in terms of biocompatibility, structural diversity, and synthesis cost, their assembly behavior is generally more sensitive to environmental conditions compared to other biomolecular strategies. A relevant benchmark in this context is DNA‐templated NP assembly.^[^ ^69^ ^]^ DNA offers unmatched structural precision thanks to the predictable nature of base pairing, allowing for consistent NP positioning and highly reproducible architectures. This contrast highlights the trade‐off between the flexibility of peptide scaffolds and the programmability of DNA. Importantly, integrating design principles from DNA nanotechnology, such as sequence‐specific binding and predictable folding, could substantially improve the reliability of peptide‐guided assemblies.

Peptide‐AuNP systems hold significant promise for applications in biomedicine and sensing, where precise control over nanoparticle structure and composition is essential. The precise spatial organization achievable through peptide‐directed assembly allows the construction of chiral plasmonic nanostructures with enhanced optical properties, already employed in biomolecular chirality detection, enantioselective sensing, and asymmetric catalysis.^[^ ^69^, ^70^ ^]^ Rational peptide design also enables the fine‐tuning of NP shape and size for biomedical uses such as photothermal therapy and advanced imaging, where these features govern optical absorption and scattering. Furthermore, highly ordered peptide‐AuNP arrays could be extremely attractive platforms for surface‐enhanced Raman spectroscopy (SERS), refractive index sensing, and diagnostic devices, benefitting from the biocompatibility and chemical versatility of peptide scaffolds.^[^ ^71^ ^]^

Key challenges must be addressed, including the reproducibility and scalability of peptide‐based assemblies and their stability under physiological conditions, to advance these technologies beyond proof of concept. Discrete nano‐assemblies offer high precision but are often difficult to scale up or integrate into functional devices. Hydrogel‐based systems are more readily processed in bulk but require strict control of diffusion and reaction conditions to ensure uniformity. Advances in peptide chemistry, such as the use of non‐natural amino acids and responsive motifs, may help overcome these limitations by expanding the functional and adaptive capacity of peptide‐AuNP constructs. Computational design approaches, including artificial intelligence and machine learning, are further accelerating development by enabling predictive modeling of peptide sequences and assembly behaviors, reducing the need for empirical screening.

In conclusion, peptide‐guided assembly of AuNPs represents a highly interdisciplinary and promising strategy that bridges chemistry, nanotechnology, and computational design. Despite current limitations in uniformity and scalability, the integration of experimental innovation with data‐driven approaches is setting the stage for practical applications in diagnostics, therapeutics, and advanced materials.

## Conflict of Interest

The authors declare no conflict of interest.
